# Single center study investigating the clinical association of donor-derived cell-free DNA with acute outcomes in lung transplantation

**DOI:** 10.3389/frtra.2023.1339814

**Published:** 2024-01-11

**Authors:** Kentaro Noda, Mark E. Snyder, Qingyong Xu, David Peters, John F. McDyer, Adriana Zeevi, Pablo G. Sanchez

**Affiliations:** ^1^ Division of Lung Transplant and Lung Failure, Department of Cardiothoracic Surgery, University of Pittsburgh, Pittsburgh, PA, United States; ^2^ Division of Pulmonary, Allergy and Critical Care Medicine, Department of Medicine, University of Pittsburgh, Pittsburgh, PA, United States; ^3^ Department of Pathology, University of Pittsburgh, Pittsburgh, PA, United States; ^4^ Departments of Obstetrics, Gynecology and Reproductive Sciences, Human Genetics and Psychiatry, University of Pittsburgh, Pittsburgh, PA, United States

**Keywords:** lung transplantation, donor-derived cell-free DNA, primary grafts dysfunction, acute cellular rejection, antibody-mediated rejection

## Abstract

**Background:**

Circulating donor-derived cell-free DNA (dd-cfDNA) levels have been proposed as a potential tool for the diagnosis of graft injury. In this study, we prospectively investigated dd-cfDNA plasma levels and their association with severe primary graft dysfunction (PGD) and graft rejection after lung transplant.

**Methods:**

A total of 40 subjects undergoing *de-novo* lung transplants at our institution were recruited in this study. Blood samples were collected at various time points before and after lung transplant for 1 year. Dd-cfDNA in samples was determined using AlloSure assay (CareDx Inc.). The correlation of the value of %dd-cfDNA was investigated with the incidence of PGD, acute cellular rejection (ACR), and donor-specific antibody.

**Results:**

We observed a rapid increase of %dd-cfDNA in the blood of recipients after lung transplantation compared to baseline. The levels of dd-cfDNA decreased during the first two weeks. The peak was observed within 72 h after transplantation. The peak values of %dd-cfDNA varied among subjects and did not correlate with severe PGD incidence. We observed an association between levels of %dd-cfDNA from blood collected at the time of transbronchial biopsy and the histological diagnosis of ACR at 3 weeks.

**Conclusion:**

Our data show that circulating dd-cfDNA levels are associated with ACR early after transplantation but not with severe PGD. Plasma levels of dd-cfDNA may be a less invasive tool to estimate graft rejection after lung transplantation however larger studies are still necessary to better identify thresholds.

## Introduction

Currently, most transplant centers monitor lung allograft quality through a combination of longitudinal transbronchial biopsies (TBBx) and pulmonary functional testing. However, given the heterogenous nature of acute cellular rejection, clinical needs for new donor graft diagnostic tools exist to know the posttransplant graft conditions and rejection more accurately and quantitatively in a non-invasive or less-invasive manner compared to the current standard practice in lung transplantation (1, 2). Liquid biopsy is a less invasive means to potentially measure graft damage, however, the donor-specific damage markers have not been found out with demonstrating their clinical relevancy and direct association with allograft rejection or dysfunction in the acute or chronic phase (3).

Cell-free DNA (cfDNA) has been reported as a biomarker of graft injury and cell death in general. Under homeostatic conditions, healthy individuals have a low abundance of circulating cfDNA; acute/chronic inflammatory diseases resulting in increased cell turnover leads to an increased abundance of circulating cfDNA (4). The half-life of cfDNA is typically less than 1–2 h, thus it's detection in the circulation can be considered a time-sensitive biomarker of a disease state (5). By differentiating donor and recipient-specific single nucleotide polymorphisms (SNPs) it is possible to determine the origin of circulating cfDNA (6). This methodology has been proposed to detect circulating donor-derived cfDNA (dd-cfDNA) as a potential tool for the identification of graft injury (rejection, infection, ischemia/reperfusion injury) (7). The proportion of circulating dd-cfDNA has been associated with the presence of allograft rejection after solid organ transplantation. In lung transplantation, there are some reports demonstrating the association of dd-cfDNA with allograft rejection, infection and primary graft dysfunction (PGD) (7–10). While previous reports utilized whole genome sequencing or targeted sequencing, the next generation of targeted gene sequencing assays have advantages in with accuracy, detection time, cost, and no requirement of donor genotype data (6, 11). The clinical grade of the next generation of targeted gene sequencing assay has already been tested for plasma biorepository samples of lung transplant patients and found the detected %dd-cfDNA was associated with allograft rejection (12–14). In this study, we prospectively investigated dd-cfDNA levels in recipients' plasma and their association with severe PGD at 72 h and acute allograft rejection after lung transplantation, using a clinically approved next generation of targeted gene sequencing assay.

## Materials and methods

### Study design

**Figure 1:**
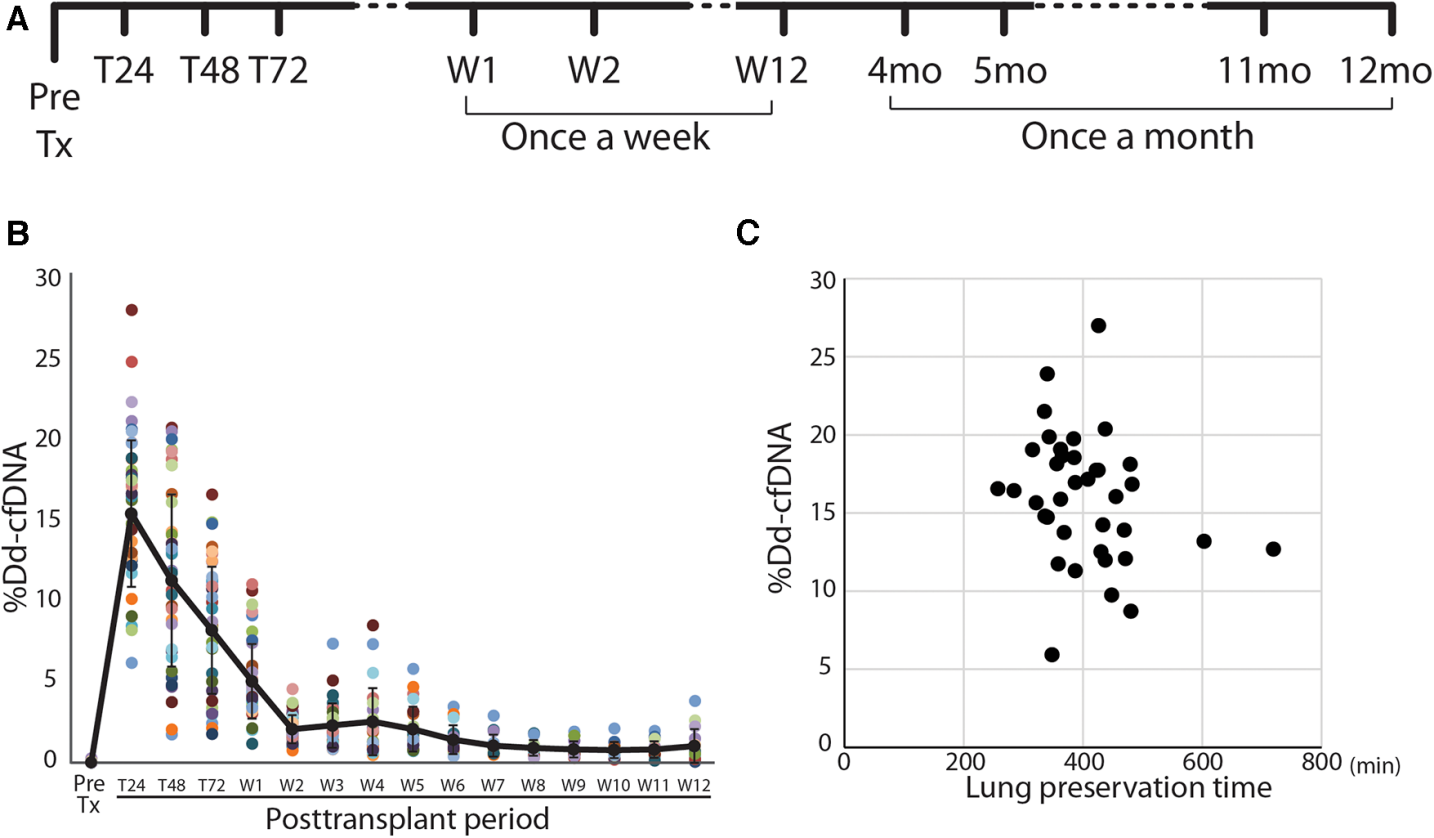
Study design and posttransplant time course of %dd-cfDNA in lung transplant patients. (**A**) The protocol and sampling plan in this study. (**B**) Time course of the percentage of donor-derived cell-free DNA (%dd-cfDNA) in the plasma of lung transplant recipients. Each patient is a color-coded circle. The black solid line shows the trend of mean %dd-cfDNA and the data are shown as mean ± SD. (**C**) The association between the lung preservation time and the peak %dd-cfDNA.

This was a single-center prospective observational study performed under the approval of the Institutional Review Board of the University of Pittsburgh (STUDY 19090098 approval: 12/2019; ClinicalTrials.gov Identifier: NCT04318587). Adults (>18 years-old, both male and female) undergoing *de-novo* lung transplantation at our institute were recruited in this study. The patients who had multi-organ transplants, bone-marrow transplants, and re-transplantation were excluded due to the detection mechanism of dd-cfDNA. All subjects were consented to the study before the transplantation. The blood samples from the consented subjects were obtained using a blood tube preserving the cfDNA (Cell-Free DNA BCT®; Streck Corporate, La Vista, NE) and scheduled to draw at pre-operation and postoperative day 1, 2, 3, once a week for 1–12 weeks, and once a month from 4 months to 12 months posttransplant (Figure 1A). After the subjects were discharged, the blood was drawn at the time of their follow-up visit.

### Determination of dd-cfDNA

The dd-cfDNA were measured using the AlloSure assay (CareDx, Inc., Brisbane, CA). Allosure assay is a clinical-grade, next generation of targeted gene sequencing assay to measure SNPs to accurately quantify dd-cfDNA. All blood samples were shipped to the CareDx facility for the AlloSure assay. The dd-cfDNA data were provided as a percentage (%dd-cfDNA) of the total (donor + recipient) cfDNA.

### Primary outcomes

The primary outcomes include the PGD grade, acute cellular rejection (ACR), and development of *de-novo* donor-specific antibody (DSA). The PGD grade for the first posttransplant 3 days was determined daily by the treating team, using the ISHLT criteria (15). Our protocol includes a surveillance bronchoscopy with TBBx at 2–3 weeks post-transplant. ACR was diagnosed and graded by a pulmonary pathologist after evaluation of histopathology slides (16).

After the subject discharging, allograft ACR was diagnosed by TBBx at the time of the subject's follow-up visit and was graded. At the same time as TBBx, blood was sampled for ddcfDNA for AlloSure assay and detecting DSA by the UPMC Histocompatibility Laboratory (17). Positive DSA was determined with mean fluorescence intensity >1,000 in a single-antigen beads assay (LABScreen class I and II, One Lambda, West Hills, CA). We analyzed the %dd-cfDNA data to see the correlation with the ACR grade and the existence of *de-novo* DSA at the same time point.

### Statistical analysis

Univariable analyses were performed using SPSS (v. 28.0, IBM, Armonk, NY). Results are shown as mean ± standard deviation (SD) with individual values or median and interquartile range (IQR). Univariable comparisons were performed using chi-square tests for categorical variables and Mann-Whitney U tests for continuous variables. Data with multiple groups were analyzed using one-way analysis of variance followed by *post hoc* analysis with the Tukey test for multiple comparisons. Multiple observations over time were analyzed using a two-way ANOVA mixed model. A probability level of *p *< 0.05 was considered statistically significant.

## Results

### Subject characteristics and posttransplant data

The patient characteristics and posttransplant data are summarized in Table 1. Forty patients (39 double lung transplants and 1 single lung transplant) were enrolled. All recipients were transplanted with grafts from brain death donors. 39 sequential double lung transplants and 1 single lung transplant were performed. One case used *ex-vivo* lung perfusion to evaluate a graft before transplant. The severe PGD incidence at 72 h posttransplant was 27.5% (*n* = 11). The mean duration of intensive care unit and hospital stay were 10.7 ± 12.9 and 27.3 ± 16.2 days, respectively (Table 2).

**Table 1:** Recipients and donors’ characteristics.

	Total	No ACR	ACR1 > within a year
Subject, *n*	40	15	25
Recipient characteristics			
Recipient age [years-old], median (IQR)	61.5 (15.3)	61.0 (15.0)	65.0 (9.0)
Recipient female, *n* (%)	17 (42.5)	11 (73.3)	6 (24.0)
LAS, median (IQR)	41.1 (10.5)	41.2 (5.1)	37.6 (11.3)
Recipient BMI (kg/m^2^), median (IQR)	26.2 (6.3)	26.7 (7.1)	26.2 (5.7)
Recipient Diagnosis, *n* (%)			
Obstructive	11 (27.5)	2 (13.3)	9 (36.0)
Pulmonary vascular	1 (2.5)	1 (6.7)	0
Suppurative	3 (7.5)	1 (6.7)	2 (8.0)
Restrictive	25 (62.5)	11 (73.3)	14 (56.0)
Donor characteristics			
Donor age [years-old], median (IQR)	34.5 (16.0)	37.0 (20.5)	32.0 (14.0)
Brain death donors, *n* (%)	40 (100)	15 (100)	25 (100)
Donor BMI [kg/m^2^], median (IQR)	25.7 (7.7)	27.1 (8.4)	24.1 (6.9)
*ex vivo* lung perfusion, *n* (%)	1 (2.5)	1 (6.7)	0
Perioperative parameters			
Total preservation time [min], median (IQR)	387.0 (83.0)	408.0 (82.5)	385.0 (91.0)
Induction, *n* (%)			
Simulect	27 (67.5)	11 (73.3)	16 (64.0)
Campath	13 (32.5)	4 (26.7)	9 (36.0)
Intraoperative support, *n* (%)			
CPB	23 (57.5)	10 (66.7)	13 (52.0)
ECMO	12 (30.0)	4 (26.7)	8 (32.0)
None	5 (12.5)	1 (6.7)	4 (16.0)
Intraoperative product volume (units), median (IQR)	7.0 (8.3)	7.0 (8.5)	7.0 (7.0)

BMI, body mass index; CPB, cardiopulmonary bypass; ECMO, extracorporeal membrane oxygenation; LAS, lung allocation score.

**Table 2:** Post-transplant data.

Double lung transplant, *n* (%)	39 (97.5)
PGD3 at 24 h, *n* (%)	8 (20.0)
PGD3 at 48 h, *n* (%)	10 (25.0)
PGD3 at 72 h, *n* (%)	11 (27.5)
CRRT within 1 week, *n* (%)	3 (7.5)
Hepatic dysfunction, *n* (%)	4 (10.0)
ICU stay, median (IQR)	5.0 (9.5)
Hospital stay, median (IQR)	22.5 (14.3)
*De-novo* DSA within a year, *n* (%)	18 (45.0)
ACR 1 > at the first TBBX, *n* (%)	13 (32.5)
ACR 1 > within a year, *n* (%)	25 (62.5)

ACR, acute cellular rejection; CRRT, continuous renal replacement therapy; DSA, donor-specific antibody; ICU, intensive care unit; PGD3, primary graft dysfunction grade 3.

### The time-dependent change of %dd-cfDNA circulating in the patient's blood after lung transplantation

A total of 515 samples were collected to determine %dd-cfDNA from the subjects. Twenty-four samples were rejected for the assay due to poor sample quality or the assay metrics issue. The time-dependent change of %dd-cfDNA in the first 3 months is shown in Figure 1B. The %dd-cfDNA in the circulation was elevated from baseline immediately after transplantation (pretransplant: mean ± SD 0.013 ± 0.05%) and showed a peak value (mean ± SD 15.5 ± 4.56%, range: 5.94–26.99%) within 48 h posttransplant. The %dd-cfDNA was decreased over 2 weeks and reached a plateau (stable) level (Figure 1B). There was no significant association between the lung allograft preservation time and the peak %dd-cfDNA (Figure 1C).

### The association of dd-cfDNA level and the PGD incidence

**Figure 2:**
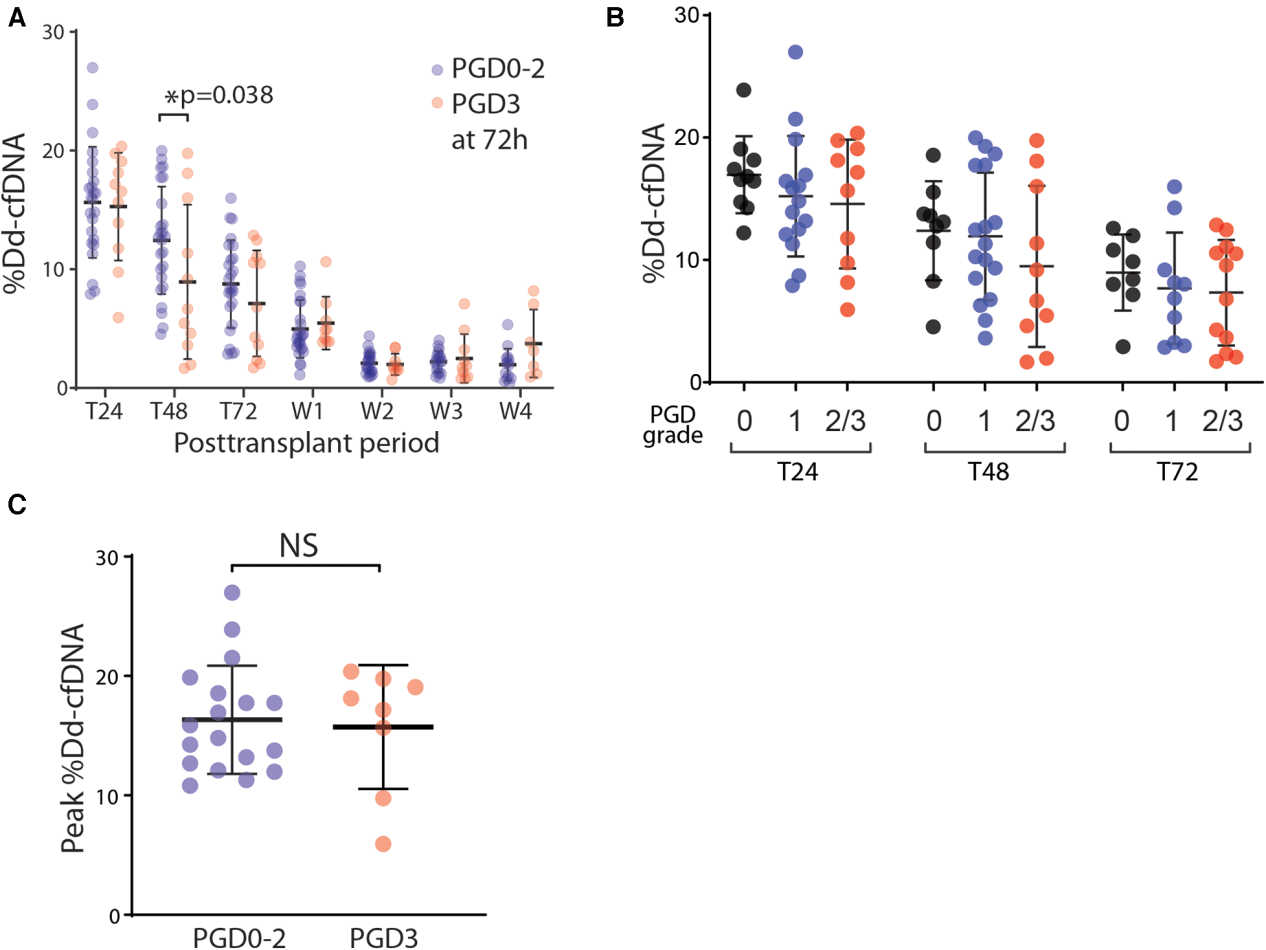
The association of PGD and the %dd-cfDNA in plasma. (**A**) The time-dependent changes of %dd-cfDNA in plasma from the lung transplant patients with primary graft dysfunction (PGD) grade 0–2 and grade 3 at 72 h posttransplant. (**B**) The correlation between the PGD grade and %dd-cfDNA in plasma for each posttransplant 24 h, 48 h and 72 h. (**C**) The peak value of %dd-cfDNA for each patient within 48 h posttransplant had no difference between the patients with/without PGD grade 3 at 72 h posttransplant. * *p *< 0.05.

We compared the circulating %dd-cfDNA between patients who developed PGD grade 3 and those who with grade 0–2, with the hypothesis that lungs with severe PGD should exhibit a greater degree of allograft cellular death, releasing a higher plasma level of dd-cfDNA. Instead, we found a lower %dd-cfDNA at T48 h in the severe PGD cohort, and no difference in %dd-cfDNA at any other timepoints up to 4 weeks (Figure 2A). We next found no difference in the %dd-cfDNA based on grade of PGD (Figure 2B). Finally, we found no statistically significant association between the peak %dd-cfDNA with the PGD3 within 24–48 h posttransplant (Figure 2C).

### The correlation of the %dd-cfDNA and graft rejection

**Figure 3:**
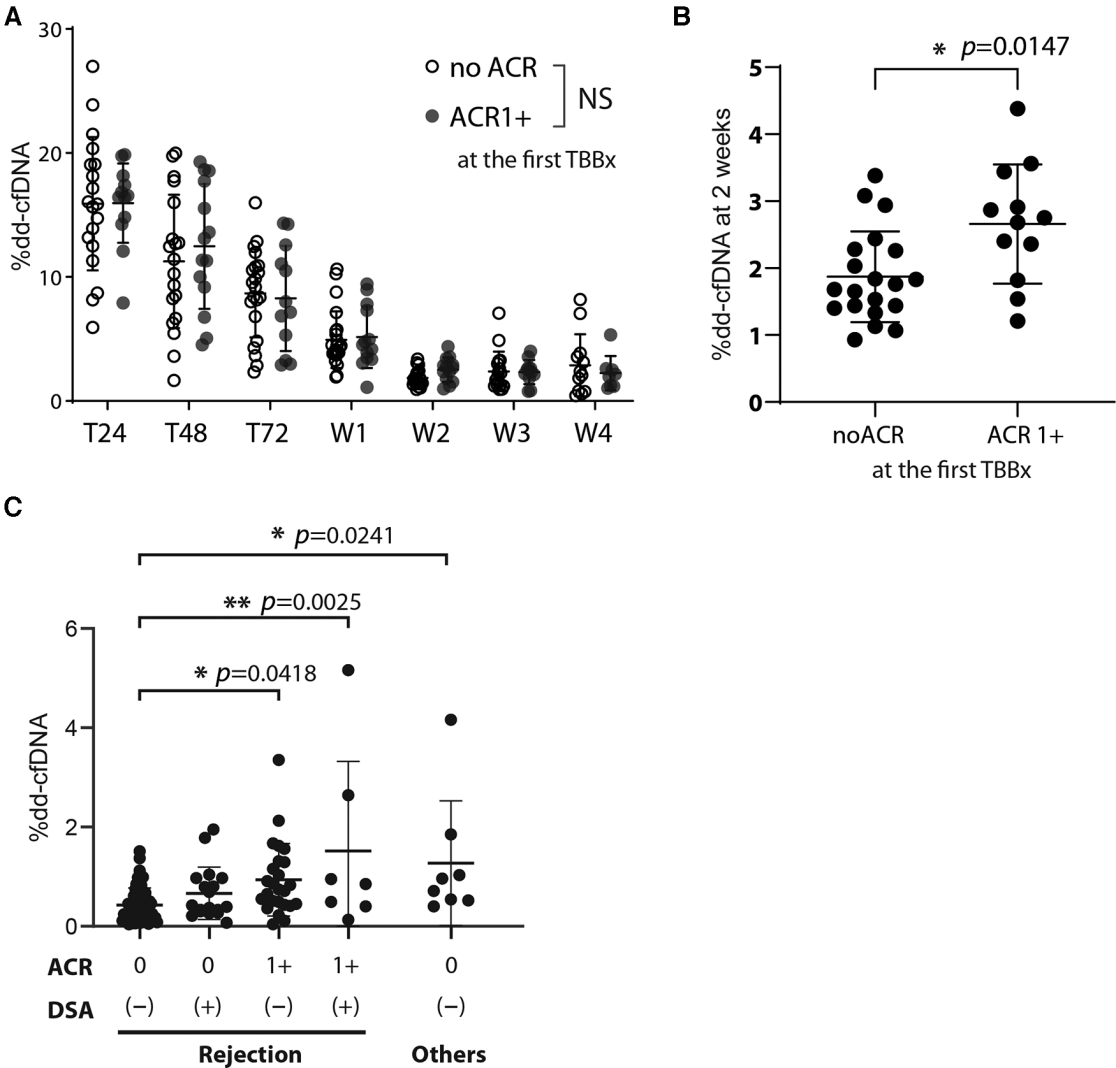
The effect of acute cellular rejection, DSA, and other injury contributors on %dd-cfDNA (**A**) the %dd-cfDNA time course for the patients who developed acute cellular rejection grade more than 1 (ACR 1+) in the posttransplant early phase (within 2–3 weeks) and diagnosed at the first TBBx, compared with that in the patients without ACR (no ACR). (**B**) Comparison of the %dd-cfDNA at 2 weeks posttransplant between the patients who did or did not exhibit ACR 1+ at the first TBBx. (**C**) The association of %dd-cfDNA at the patient follow-up visits after the hospital discharge and their results of TBBx and DSA. The factors other than rejection but could associate with %dd-cfDNA were isolated from the analysis among the rejection cluster, and their effect was analyzed separately. Other injury contributors include bronchiolitis, sepsis, anastomosis stenosis, and pseudomonas pneumonia. NS: not significant, * *p *< 0.05, ** *p *< 0.01.

At our institution, we perform the first TBBx within 2–3 weeks of transplantation to detect early ACR. We compared the %dd-cfDNA in the days to weeks after transplantation with the histopathological diagnosis of early ACR. When looking at longitudinal, timepoint matched values of circulating %dd-cfDNA, we found no statistically significant difference between those study participants who developed early ACR and those that did not (Figure 3A). However, we found a statistically significant increase in %dd-cfDNA in the plasma drawn from recipients posttransplant 2 weeks and the first TBBx showing early ACR (Figure 3B). Although statistically significant, there was substantial overlap between groups, and the upper limit of %dd-cfDNA in the early ACR group was well below levels found at 1 week after transplantation.

We next looked at the %dd-cfDNA compared to the presence of ACR and *de-novo* DSA diagnosed after discharging to 1-year follow-up period. We analyzed data to see the correlation of %dd-cfDNA with the ACR grade based on TBBx and the existence of *de-novo* DSA at the same time point. Contrary to recent reports about the association of antibody mediated rejection and %dd-cfDNA (12), we found that %dd-cfDNA were not significantly higher in patients with *de-novo* DSA (Figure 3C). However, patients with both ACR and *de-novo* DSA had a significantly higher %dd-cfDNA than those without either (Figure 3C). Interestingly, other sources of allograft injury (bronchiolitis, sepsis, anastomosis stenosis, and pseudomonas pneumonia) had comparable elevation in %dd-cfDNA as those with both ACR and *de-novo* DSA. These findings suggest that %dd-cfDNA could not differentiate sources of perioperative lung injury.

## Discussion

### Potential disturbances for %dd-cfDNA as an early biomarker

We provisionally investigated the association of %dd-cfDNA determined by the next generation of gene sequence technology with early perioperative outcomes including the PGD, ACR, and *de-novo* DSA in recipients of lung transplantation. Regardless of PGD grade, most study participants had a substantial increase in the proportion of dd-cfDNA immediately after lung transplantation. Similar results have been observed in kidney transplantation and this is considered a result of ischemia-reperfusion injury (18). While we observed the %dd-cfDNA reached the baseline level within 2 weeks and decreased at 86.5% from the peak value in this study, the trend of %dd-cfDNA could differ among the organ types. Studies in kidney transplant demonstrated that %dd-cfDNA converged to the plateau within a week (19–21), while the multicenter study “GRAfT” for heart transplants showed it reached the baseline by ∼60 days (22). Allograft cell death should correlate with grade of ischemia-reperfusion injury and PGD after solid organ transplantation (23). Our findings in lung transplant setting are not consistent with this hypothesis owing to 3 potential explanations. First is the nature of the test itself, measuring the proportion and not absolute value of the dd-cfDNA; second, is the systemic symptom of PGD3; and the third, is the unknown source of the cfDNA. As previously mentioned, the %dd-cfDNA is the ratio of dd-cfDNA among the total cfDNA circulating in the blood of recipients which is likely impacted by recipient-derived cfDNA, especially with inflammatory cell turnover. Thus, increased occupation of plasma recipient-derived cfDNA in response to systemic conditions can reduce the %dd-cfDNA proportionally (24). While %dd-cfDNA is often utilized in clinical currently rather than the absolute copy or concentration of cfDNA, the quantification of dd-cfDNA copy may represent the PGD phenotype or rejection without recipients' noise (25, 26). Since this method measures the ratio, there is no method to normalize across patient characteristics or procedure-related biases (e.g., body size, patient condition, single/double lung transplant, blood transfusion).

Severe PGD is now appreciated to be a systemic condition which may impact the proportion of donor and recipient derived cfDNA (27). Severest PGD patients are often comorbid with acute kidney failure or liver dysfunction and these lead patients to systemic illness and require additional life support therapy (28, 29). Some of our subjects with severe PGD showed critical illness in this study and required hemodialysis in addition to extracorporeal membrane oxygenation (ECMO). These factors could affect both recipient and total DNA levels and create potential bias on %dd-cfDNA through material-biological interaction, increased recipient-derived cfDNA, and elimination of DNA by hemodialysis (30–32). Further basic investigations are required to clarify the effects of these disturbances on the net cfDNA and the diagnosis power of dd-cfDNA for posttransplant graft outcomes in lung transplantation.

Another important aspect of our finding is that the spike of the %dd-cfDNA was observed in the non-PGD group as well, suggesting this cellular death is not associated with graft injury and dysfunction. undetectable cellular source of cfDNA is one of the limitations of the bulk dd-cfDNA assay, thus we could not know whether the dd-cfDNA was resulting from the tissue injury after ischemia-reperfusion, or just washing out effects of the remnant cfDNA carried over from the donors in grafts. While the vascular bed of grafts is washed by antegrade and retrograde flushing at procurement, some donor cells [aka. microchimerism (33)] and other blood components could remain in the allografts (23, 34). Therefore, the dd-cfDNA released from the allografts immediately after transplantation could contain multiple factors, and determining the source of the dd-cfDNA within the first 3 days is of interest to define its association with the allograft damage and increase the graft specificity (35).

### Acute graft rejection and %dd-cfDNA

We observed significantly increased %dd-cfDNA was associated with ACR alone and combined allograft rejection (ACR + *de-novo* DSA), but not *de-novo* DSA alone. Despite these statistically significant increases in %dd-cfDNA with both ACR and combined rejection, the percentage point increases were small; the majority had less than 2% in most cases. This modest increase in %dd-cfDNA does not provide us much insight into the relationship between graft cell death and the alloreactive response. It is entirely possible that rate of graft cell death occurs much more slowly in the setting of allorecognition (36). Alternatively, the low proportion of %dd-cfDNA in the setting of rejection represents a concomitant increase in circulating cfDNA of both donor graft cell death and recipient-derived immune cell turnover, leading to variable and modest changes in the circulating %dd-cfDNA. As mentioned, the lifetime of cfDNA in blood circulation is usually 1–2 h, thus it is hard to capture a slow-progressing cellular death in grafts by detecting dd-cfDNA. Multi-factorial rejection may enhance cell death in grafts thus the %dd-cfDNA has responded higher in the mixed allograft damage than the single factor rejection.

Ultimately, the limited range of circulating %dd-cfDNA in the setting of early allograft rejection raises questions about the ability of circulating %dd-cfDNA to identify specific patterns of allograft injury with any degree of resolution and sensitivity. This poor resolution as a diagnostic tool has been reported in other solid organ transplants, with levels range above 1.0% of dd-cfDNA for a risk of active rejection, especially above 0.5% for lung transplantation (37). Not only the graft rejection, the %dd-cfDNA in lung transplantation could correlate with exogenous and endogenous effectors, such as aspiration, bronchiolitis, infection, or malignancy, which augment mass cell death. Thus, the %dd-cfDNA can supplementally be important to support diagnosing graft rejection with TBBx, DSA detection, or other conventional tools. More sophisticated means of determining the cellular origin of circulating cfDNA used in concert with other metrices may provide the detection of dd-cfDNA the granularity needed to be a clinical useful biomarker. However, as it remains now, the %dd-cfDNA does not provide the precision necessary to justify broad clinical utilization.

### Limitations

There are several limitations of dd-cfDNA study in general. Without performing the donor genotyping, the next generation of gene sequencing technology cannot identify the dd-cfDNA in the patient plasma after re-transplantation, multi-organ transplantation, and bone marrow Tx case. In addition, we were not able to get the %dd-cfDNA data for 24 samples out of 515 samples, because of the sample quality and the assay quality metrics issue. One of the biggest issues in the sample quality is hemolysis and hemolysis can happen with improper sampling in general or a longer sample storage in the tube [>5 days (38)], or it may be originated in the patients due to ECMO, cardiopulmonary bypass, blood transfusion or hemodialysis. This may result in an increase in the background recipient-derived cfDNA, thus those samples were rejected from the assay (39).

## Conclusion

Our data show that circulating %dd-cfDNA did not show a relevant association with PGD, suggesting potential room to refine this technology in order to become a better biomarker for acute graft injury. Plasma levels of dd-cfDNA may be a less invasive tool to estimate graft rejection after lung transplantation however larger studies are still necessary to better identify thresholds.

## Data Availability

The raw data supporting the conclusions of this article will be made available by the authors, without undue reservation.
